# The effect of intracanal medicaments used in Endodontics on the dislocation resistance of two calcium silicate-based filling materials

**DOI:** 10.1186/s12903-020-1044-6

**Published:** 2020-02-18

**Authors:** Sara Alsubait, Norah Alsaad, Sumayyah Alahmari, Fatimah Alfaraj, Hussam Alfawaz, Abdullah Alqedairi

**Affiliations:** 1 Division of Endodontics, Department of Restorative Dental Science, College of Dentistry, King Saud University, Riyadh, Saudi Arabia; 2 Private Practice, Riyadh, Saudi Arabia; 3 Endodontic Residency Program, Saudi Commission for Health Specialist, Riyadh, Saudi Arabia

**Keywords:** Bond strength, Calcium hydroxide, Calcium silicate-based cements, Endodontics, Material testing, Modified triple antibiotic paste

## Abstract

**Background:**

Intracanal medicaments can be used in various endodontic conditions including multiple visit endodontics after trauma or in regenerative endodontics. These medicaments should be removed from the root canal before the placement of the filling or repair material. The aim of the present study was to evaluate the effect of prior calcium hydroxide (Ca(OH)_2_) and modified triple antibiotic paste (mTAP) placement on the push-out bond strength of TotalFill BC fast set putty (BC fast set putty) to root dentin when compared to mineral trioxide aggregate (MTA).

**Methods:**

The root canals of 45 extracted mandibular premolars were prepared to a standardized internal diameter (1.5 mm). The specimens were randomly assigned to 3 groups according to the intracanal medicament used: mTAP (a mixture of metronidazole, ciprofloxacin, and cefaclor), Ca(OH)_2_, and no intracanal medicament. After 1 week, the medicaments were removed, and the middle third of the roots were cut into two transverse sections (2.0 ± 0.05) (*n* = 90 slices). Thereafter, the specimens were divided into two subgroups (*n* = 45 each): MTA or BC putty. After 1 week, the push-out test was performed and failure mode was evaluated. The data were statistically analyzed using two-way ANOVA and Tukey’s post hoc.

**Results:**

The application of the intracanal medicament did not significantly affect the bond strength of BC putty (*p* > .05). For MTA, the prior application of Ca(OH)_2_ or mTAP significantly decreased the dislocation resistance (*p* < .05). Specimens in the MTA subgroups showed an almost equal number of cohesive and mixed types of failure while the majority of the specimens in the BC putty subgroups revealed the cohesive type.

**Conclusions:**

Ca(OH)_2_ and mTAP promoted lower bond strength of MTA to root dentin compared to the control group. However, the BC fast set putty bond strength to dentin was not affected by prior medication with Ca(OH)_2_ or mTAP.

## Background

The role of microorganisms as the primary cause of apical periodontitis is well documented in the literature [1, 2]. Therefore, endodontic treatment procedures are directed toward the elimination of microorganisms from the root canal system. Bacterial elimination is performed using chemomechanical debridement. However, this use of instrumentation and irrigation does not render the root canal free of bacteria [3]. The use of an interappointment medicament has been shown to significantly improve disinfection after chemomechanical procedures [4]. Calcium hydroxide [Ca(OH)_2_] is a commonly used intracanal medicament. It possesses several advantages such as tissue dissolving ability [5] and antibacterial properties [6]. Triple antibiotic paste (TAP) is another example of an intracanal medicament. It is composed of ciprofloxacin, metronidazole, and minocycline [7]. This mixture has been used for disinfection of the root canal system during root canal treatment and pulpal regeneration [8, 9]. The main drawback of TAP is tooth discoloration caused by minocycline [10]. Therefore, cefaclor has replaced minocycline in this paste and is described as modified triple antibiotic paste (mTAP) [11].

Calcium silicate-based cements are commonly used in endodontics for different clinical procedures including pulpal regeneration, apexification, and perforation repair [12]. Mineral trioxide aggregate (MTA), a calcium silicate-based cement, has been widely used in endodontics since its development in the 1990s [13]. MTA has several desirable properties such as biocompatibility [14] and bioactivity [15]. The main disadvantages of MTA are the long setting time [16], difficulty in handling [17], and discoloration potential [18]. TotalFill BC (FKG, Dentaire, La Chaux-de-Fonds, Switzerland; marketed as EndoSequence BC from Brasseler USA in North America) is a newer calcium silicate-based cement that was developed to overcome the disadvantages of MTA. It is a biocompatible [19] premixed cement that sets in the presence of water and has shorter setting time than MTA [16]. It is also reported that BC is a bioactive cement [20] with similar compressive strength to MTA [21]. Furthermore, it does not induce discoloration in the tooth structure [18].

After canal disinfection and the medication period, the intracanal medicament should be removed from the root canal before the placement of the filling or repair material. One of the requirements of calcium silicate cements is resistance to dislodgement when exposed to occlusal or operative procedures [22]. Previous studies reported that Ca(OH)_2_, TAP and mTAP do not affect the dislocation resistance of MTA to root dentin [23, 24]. However, Turk et al. [25] found that the application of TAP for longer than 2 weeks or Ca(OH)_2_ for longer than 4 weeks has a negative effect on MTA-dentin bond strength. To the best of our knowledge, no studies have evaluated the effects of intracanal medicaments on the dislocation resistance of BC cement to root canal dentin. Therefore, the purpose of the present study was to evaluate the effect of prior Ca(OH)_2_ and mTAP placement on the push-out bond strength of TotalFill BC root repair material fast set putty to root dentin compared to those of MTA. The null hypothesis was that intracanal medicaments would not affect the bond strength of TotalFill BC root repair material and MTA.

## Methods

This research was approved by the Institutional Review Board of King Saud University in Riyadh, Saudi Arabia (E-18-3078), and the College of Dentistry Research Center (FR0446) of King Saud University in Riyadh, Saudi Arabia. Sample size calculation was performed using G*Power 3.1.9.4 software at alpha error probability of 0.05 with effect size of 0.43 and power of 0.88. The power analysis showed that a total of 90 specimens (15 per group) were required.

### Specimen preparation

Specimens were prepared as described previously by Nagas et al. [24] with some modifications. Forty-five extracted single-rooted human mandibular premolars were selected. Teeth had been extracted for reasons unrelated to this study. The teeth were examined under dental operating microscope (Zeiss, Oberkochen, Germany) to exclude any teeth with root caries, cracks, or fractures. Teeth with one noncalcified canal, as confirmed by radiographs taken from buccolingual and mesiodistal views, were selected. Premolars with canals of more than a 20° curvature [26] were excluded. The teeth were stored in saline until used.

The teeth were embedded vertically in epoxy resin (Vertex Orthoplast; Vertex-Dental, Zeist, Netherlands). Specimens were sectioned apically 12 mm and coronally 1 mm below the cementoenamel junction using an Isomet low speed saw (Isomet; Buehler Ltd., Lake Blu, NY, USA) with continuous water irrigation. The working length was determined by placing a size 10 K-file (Maillefer, Ballaigues, Switzerland) in the canal until it was visible at the apical foramen. The glide path was established by hand filing with 15 and 20 K-file sizes to full working length. Instrumentation was completed with ProTaper rotary instruments (Dentsply Maillefer, Ballaigues, Switzerland) to the F3 file (tip size 30 and 0.09 taper) at 300 rpm. To obtain a standard parallel internal diameter of 1.5 mm, Peeso reamers (Maillefer, Ballaigues, Switzerland) between #1 and #5 were introduced in the root canals, and a #5 Peeso reamer was allowed to protrude 1 mm beyond the apex. Each instrument was replaced after preparing three canals or if any sign of deformation was observed. The root canals were irrigated using 2 mL 2.5% sodium hypochlorite (NaOCl) after each instrument. After completion of the instrumentation process, the smear layer was removed with 10 mL 17% ethylenediaminetetraacetic acid (EDTA) (Pulpdent, Watertown, USA) applied over a period of 1 min followed by 10 mL of 2.5% NaOCl. The canals were then flushed with 20 mL sterile saline to remove the residual irrigant and dried with sterile paper points (Dentsply Maillefer, Tulsa, Oklahoma, USA). A single operator prepared the root canals under dental operating microscope.

The specimens were randomly assigned to 3 groups (*n* = 15 each) with respect to the intracanal medicament used: mTAP, Ca(OH)_2_, and the control group. mTAP was prepared by mixing equal portions of metronidazole (Eczacibasıbası, Istanbul, Turkey), ciprofloxacin (Bayer, Leverkusen, Germany), and cefaclor (Sanovel, Istanbul, Turkey) with sterile distilled water (powder/liquid ratio of 3:1) [27]. UltraCal XS (Ultradent, South Jordan, UT) was used for the Ca(OH)_2_ group. No intracanal medicament was placed in the control group. The medicament was delivered into the canals using a lentulo spiral (Dentsply Maillefer, Ballaigues, Switzerland) in a slow-speed handpiece and tamped down in the canal space using the blunt ends of sterile paper points. The apical root canal and coronal orifices were sealed with glass ionomer cement (Fuji, GC, Tokyo, Japan). The specimens were stored in an incubator at 95% humidity and 37 °C. After 1 week, the temporary filling material was removed with a round bur (Dentsply Maillefer) under water cooling and followed by the removal of the medicaments using 2 mL of 2.5% NaOCl, 17% EDTA, and sterile saline. Then, the root canals were dried using absorbent paper points. The middle third of each root was cut into two 2.0 ± 0.05 parallel transverse sections (*n* = 90 slices) in the coronal-to-apical direction using an Isomet low speed saw with continuous water irrigation. Thereafter, the specimens were divided into two subgroups (*n* = 45 each) according to the calcium silicate cement applied: (i) MTA (ProRoot MTA, Dentsply Tulsa Dental, Tulsa, OK, USA) or (ii) Totalfill Root Repair Material - fast set putty. MTA was mixed according to manufacturer’s instructions. Totalfill Root Repair Material - fast set putty (BC fast set putty) is a premixed ready to use cement. The cements were placed inside the lumen of the slices and condensed using an endodontic plugger on a flat glass plate. Then, a scalpel was used to remove the excess material. Finally, the specimens were wrapped in pieces of gauze soaked in distilled water and stored in an incubator for 1 week.

### Push-out bond strength test

The push-out test was conducted using a universal testing machine (Instron testing machine; Model 5965, ITW, MA, USA). The samples were placed on a metal slap containing a central hole to allow free motion of the plunger. Loading on the calcium silicate cement material was applied using a 1.2 mm-diameter plunger at a constant vertical pressure and a speed of 1 mm/min until total bond failure occurred. The plunger tip was positioned to contact the test material only. The highest force applied to materials at the time of dislodgement was recorded in megapascal (MPa) by using the following formula:

$$ Bond\ strength\ (MPa)=\frac{\mathrm{Force}\ \mathrm{for}\ \mathrm{dislodgement}\ \left(\mathrm{N}\right)}{\mathrm{Bonded}\ \mathrm{surface}\ \mathrm{area}\ \left({mm}^2\right)} $$

$$ \mathrm{Bonded}\ \mathrm{surface}\ \mathrm{area}=2\times 3.14\times \mathrm{radius}\ \mathrm{of}\ \mathrm{the}\ \mathrm{root}\ \mathrm{canal}\times \mathrm{the}\ \mathrm{thickness}\ \mathrm{of}\ \mathrm{dentin}\ \mathrm{slice} $$

The operator who made the measurements was blinded to which sample was matched to which material.

### Evaluation of failure patterns

The failure mode in each slice was identified under a digital microscope at 50X magnification (KH-7700, Hirox Co, Tokyo, Japan); these included adhesive failure that occurred at the dentin-material interface, cohesive failure that occurred within the material, or mixed failure, which is the combination of the two failure modes. The operator who examined the slices was blinded to which sample was matched to which material.

### Statistical analysis

Statistical analysis was performed using the Statistical Package for the Social Sciences statistical software (version 21.0, SPSS IBM, Armonk, NY). The data were normally distributed according to Kolmogorov-Smirnov. Then, data were statistically analyzed using two-way analysis of variance (ANOVA). When applicable, Tukey’s post hoc was performed for multiple comparisons. The chi-squared test was used to determine if there was a significant association between the type of failure and the filling material or intracanal medicament. The statistical significance was set at 0.05.

## Results

### Push-out test

The mean and standard deviations of the push-out bond strength values are presented in Table 1. Two-way ANOVA indicated no significant difference between MTA and BC fast set putty regardless the use of intracanal medicament (*p* = .20). For both cements, the highest push-out bond strength results were obtained without prior medication (control). However, the application of intracanal medicament did not significantly affect the bond strength of BC fast set putty (*p* = .63). For MTA, prior application of Ca(OH)_2_ or mTAP significantly decreased the dislocation resistance (*p* = .00 and *p* = .00, respectively) with similar debonding values for both medicaments (*p* = .61).

**Table 1:** Means and standard deviations for push-out bond strength values (MPa) of different calcium silicate cements with respect to intracanal medicaments

	Intracanal Medicaments			*P*-value
	Control	Ca(OH)_2_	mTAP	
MTA	88.14 ± 33.43^a,A^	58.81 ± 16.28^b,A^	50.76 ± 9.65^b,A^	0.00
BC fast set putty	78.18 ± 11.65^a,A^	69.19 ± 37.47^a,A^	70.85 ± 23.34^a,B^	0.63
*P*-value	0.31	0.35	0.01	

MTA, ProRoot mineral trioxide aggregate; BC fast set putty, TotalFill Root Repair Material fast set putty; Ca(OH)_2_, Calcium hydroxide; mTAP, modified triple antibiotic paste. Different superscripted lowercase letters indicate statistically significant differences in dislodgement resistance between the control, Ca(OH)_2_, and mTAP groups within each material (*p* < .05). Different superscripted uppercase letters indicate statistically significant differences in dislodgement resistance between MTA and BC fast set putty within each intracanal medicament group (*p* < .05)

Without prior medication (control) or with prior application of Ca(OH)_2_, there was no significant difference in bond strength between MTA and BC fast set putty (*p* = .31 and *p* = .35, respectively). With prior application of mTAP, the BC fast set putty showed significantly higher debonding forces than MTA (*p* = .01).

### Bond failure patterns

**Fig. 1:**
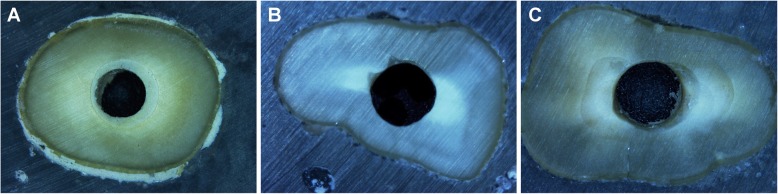
Microscopic representative images (50X magnification) showing (**a**) cohesive, (**b**) adhesive, and (**c**) mixed bond failure

Representative microscopic images of the modes of failure are shown in Fig. 1. Few specimens revealed an adhesive type of failure (Table 2). Most specimens in the MTA subgroups showed a nearly equal number of specimens in the cohesive and mixed types while the majority of specimens in all BC fast set putty subgroups revealed the cohesive type of failure. There was no significant association between the type of cement or the intracanal medicament and type of bond failure (*p* = .07 and *p* = .81, respectively).

**Table 2:** Percentage of different modes of failure in each subgroup

Mode of failure % (n)	MTA			BC fast set putty		
	Control	Ca(OH)_2_	mTAP	Control	Ca(OH)_2_	mTAP
Adhesive	13.33 (2)	6.67 (1)	13.33 (2)	0 (0)	13.33 (2)	13.33 (2)
Cohesion	46.67 (7)	46.67 (7)	40 (6)	73.33 (11)	60 (9)	66.67 (10)
Mixed	40 (6)	46.67 (7)	46.67 (7)	26.67 (4)	26.67 (4)	20 (3)

MTA ProRoot mineral trioxide aggregate, BC fast set putty, TotalFill Root Repair Material fast set putty; Ca(OH)_*2*_ Calcium hydroxide, mTAP Modified triple antibiotic paste

## Discussion

Intracanal medicaments can be used in various endodontic conditions including multiple visit endodontics after trauma or in regenerative endodontics [28]. These medicaments can be applied for different periods varying from 1 week to several months [29]. The American Association of Endodontists recommends the application of intracanal medicament for a minimum of 1 week for clinical regenerative procedures [30]. Furthermore, it has been reported that a 7-day interappointment dressing with Ca(OH)_2_ was sufficient to reduce intraradicular bacteria to a level that provided a negative culture [31]. However, the long-term exposure to interappointment medicament has been suggested to affect the mechanical properties of root dentin by either collagen degradation in the case of Ca(OH)_2_ or excessive demineralization in the case of antibiotic pastes that might affect the root fracture resistance [32]. Interim medicaments should be removed before the placement of filling/repair material. Previous studies reported that there is no method (so far) is able to completely remove intracanal medicaments from the root canal system [33, 34]. Therefore, this study aimed to evaluate the effect of Ca(OH)_2_ and mTAP after short-term application on BC fast set putty in comparison to MTA. Calcium silicate cements might be exposed to dislodging forces when used for perforation repair, apexification or regenerative procedures. One of the requirements of these cements is the ability to persist in place under dislodging forces [22]. The push-out test has been used in previous studies to assess the bond strength of calcium silicate cements to dentin [24, 29]. This method, which was used in this study, has several advantages including practicality and reliability [22].

The results obtained from this study reveal that regardless of the use of intracanal medicament, there was no significant difference in the bond strength between MTA and BC fast set putty. This result is in line with an earlier study, which reported that there is no significant difference in the dislocation of both materials [35]. Furthermore, the prior application of Ca(OH)_2_ and mTAP were associated with a significant decrease in the debonding force of MTA. This significant decrease might be explained by the degradation effect of Ca(OH)_2_ on the dentin collagen matrix caused by the low molecular weight, alkaline pH [36] and the demineralization effect of antibiotic pastes on dentin because of their acidic nature [32]. The effect of mTAP on the dentin mineral contents was investigated in a recent study and it has been demonstrated that it causes a significant increase in calcium and the calcium/phosphorus ratio [37]. Topcuoglu et al. [23] found that the prior application of Ca(OH)_2_ and mTAP do not affect the MTA dislodgement resistance. This discrepancy may be related to the differences in experimental procedures. Topcuoglu et al. [23] used NaOCl and distilled water to remove the intracanal medicament while NaOCl followed by EDTA and saline were used in the present study. The use of a chelating agent after application of intracanal medicament may cause alteration in the chemical structure of the dentin surface and surface properties that may affect material adhesion to dentin [38].

Compared with the control, the tested intracanal medicaments did not affect the BC fast set putty bond strength. In the literature, there is no data about the effect of various canal dressings on the dislocation resistance of BC fast set putty to root dentin. Therefore, the results of this study were compared with a study in which the effects of various medicaments on the dislocation resistance of BC sealer was evaluated. Gokturk et al. [39] found that the application of Ca(OH)_2_ or an antibiotic paste, which is composed of ciprofloxacin and metronidazole, did not affect the dislodgement resistance of the BC sealer.

The present study demonstrated that the highest push-out bond strength values in both groups were obtained without prior medication. This might be explained by the medicament residue in the canal walls that could act as a barrier and interfere with the material adaptation with dentinal tubules. MTA and BC fast set putty belong to a calcium silicate-based group of materials. During the hydration reaction, endodontic bioceramics release Ca(OH)_2_, which interacts with phosphates in the tissue fluids to form hydroxyapatite and tag-like structures in dentin [40]. BC fast set putty is premixed by the manufacturer and has a more homogenous and smaller particle size than MTA. Smaller particles might result in easier and deeper penetration into dentinal tubules even in the presence of medicament residue in the canal walls. This may explain the nonsignificant decrease in the debonding force of BC fast set putty compared to the significant decrease in the MTA group.

All specimens were evaluated after the push-out test to determine the mode of failure. Most failures observed in the MTA subgroups were either cohesive or mixed types. This finding is in line with previous studies [23, 29]. The bond failure observed in all BC fast set putty subgroups was predominantly within the material itself. This mode of failure may be explained by the smaller and uniform size of particles in the BC fast set putty, which enabled better penetration into the dentinal tubules and resulted in better adhesion. This finding contrasts with the results of Kadic et al. [41] who reported that BC-dentin bond failures were usually the mixed type. The conflicting results could be due to the different factors included in the study designs; slices were prepared from the apical third of the root in the Kadic et al. [41] study instead of the middle root slices used in the present study. Variations in both the number and size of dentinal tubules in the different root canal portions have been reported in the literature [42]. Furthermore, their sections were prepared after material placement inside the root canal that might affect the material adaptation.

Within the limitations of this study, the results might provide information that can aid the clinician in the selection of the best material used in clinical practice. Based on our findings, the dislocation resistance of BC fast set putty is not affected by the prior application of Ca(OH)_2_ or mTAP. Considering its additional advantages, including ease in handling and that it does not induce color changes in tooth structure, BC fast set putty might be a suitable replacement for MTA after the use of such intracanal medicament to disinfect the root canal, for example, in regenerative endodontics or apexification procedures. However, further experiments are needed to evaluate the effect of intracanal medicaments on other physiochemical properties of BC fast set putty.

## Conclusion

Within the conditions of the present study, it can be concluded that Ca(OH)_2_ and mTAP promoted lower bond strength of MTA to root dentin compared to the control group. However, the BC fast set putty bond strength to dentin was not affected by prior medication with Ca(OH)_2_ or mTAP.

## Data Availability

The datasets for the current study are available from the corresponding author on reasonable request.

## Abbreviations

ANOVA: Analysis of variance
BC fast set putty: TotalFill BC fast set putty
Ca(OH)_2_: Calcium hydroxide
EDTA: Ethylenediaminetetraacetic acid
MPa: Megapascal
MTA: Mineral trioxide aggregate
mTAP: Modified triple antibiotic paste
NaOCl: Sodium hypochlorite
TAP: Triple antibiotic paste
